# Androgen deprivation therapy increases the sensitivity of human prostate carcinoma cells to T cell-mediated lysis through an androgen receptor dependent mechanism

**DOI:** 10.1186/2051-1426-2-S3-P183

**Published:** 2014-11-06

**Authors:** Sofia Gameiro, Andressa Ardiani, Anna R Kwilas, Renee N Donahue, James W Hodge

**Affiliations:** 1 NCI/CCR/LTIB, Bethesda, MD, USA

## 

Despite recent advances in diagnosis and therapy, prostate cancer remains the most frequently diagnosed non-skin cancer in the United States and the third leading cause of cancer deaths. Failure of chemotherapies and hormone-deprivation therapies is the major cause of death in patients with castration-resistant prostate cancer (CRPC). Currently, the androgen inhibitors Enzalutamide and Abiraterone are approved for treatment of metastatic CRPC. Enzalutamide is a second-generation androgen antagonist with no agonist activity. Abiraterone is a cytochrome P450 inhibitor that blocks adrenal and intratumoral androgen production. Here we show for the first time that both Enzalutamide and Abiraterone induce immunogenic modulation in human prostate tumor cells, rendering them more sensitive to T cell-mediated lysis, and that these immunomodulatory activities are androgen receptor (AR)-dependent. In studies reported here, exposure in vitro and in vivo of human prostate carcinoma cells to Enzalutamide significantly down-regulated the anti-apoptotic NAIP gene. Functional analysis revealed that NAIP played a critical role in inducing CTL sensitivity. Amplification of AR is a major mechanism of resistance to androgen-deprivation therapy (ADT). Here, we show that Enzalutamide enhances sensitivity to immune-mediated killing of prostate tumor cells that overexpress AR. The immunomodulatory properties of Enzalutamide and Abiraterone provide a rationale for their use in combination with immunotherapeutic agents in CRPC, particularly for patients with minimal response to Enzalutamide or Abiraterone alone, or for patients who have developed resistance to ADT.

